# Maternity Care Preferences for Future Pregnancies Among United States Childbearers: The Impacts of COVID-19

**DOI:** 10.3389/fsoc.2021.611407

**Published:** 2021-02-18

**Authors:** Theresa E. Gildner, Zaneta M. Thayer

**Affiliations:** ^1^ Department of Anthropology, Dartmouth College, Hanover, NH, United States; ^2^ Department of Anthropology, Washington University in St. Louis, St. Louis, MO, United States; ^3^ Ecology, Evolution, Environment and Society Program, Dartmouth College, Hanover, NH, United States

**Keywords:** out of hospital, homebirth, birth center, midwife, barriers to care

## Abstract

The COVID-19 pandemic has impacted maternity care decisions, including plans to change providers or delivery location due to pandemic-related restrictions and fears. A relatively unexplored question, however, is how the pandemic may shape future maternity care preferences post-pandemic. Here, we use data collected from an online convenience survey of 980 women living in the United States to evaluate how and why the pandemic has affected women’s future care preferences. We hypothesize that while the majority of women will express a continued interest in hospital birth and OB/GYN care due to perceived safety of medicalized birth, a subset of women will express a new interest in out-of-hospital or “community” care in future pregnancies. However, factors such as local provider and facility availability, insurance coverage, and out-of-pocket cost could limit access to such future preferred care options. Among our predominately white, educated, and high-income sample, a total of 58 participants (5.9% of the sample) reported a novel preference for community care during future pregnancies. While the pandemic prompted the exploration of non-hospital options, the reasons women preferred community care were mostly consistent with factors described in pre-pandemic studies, (e.g. a preference for a natural birth model and a desire for more person-centered care). However, a relatively high percentage (34.5%) of participants with novel preference for community care indicated that they expected limitations in their ability to access these services. These findings highlight how the pandemic has potentially influenced maternity care preferences, with implications for how providers and policy makers should anticipate and respond to future care needs.

## Introduction: Factors Shaping Maternity Care Preferences and Barriers to Preferred Care

Several factors are known to impact maternity care preferences among women living in the United States, particularly concerns about safety and risk during delivery (Klein et al., 2006; Miller and Shriver, 2012). There is a general cultural perception that technology-intensive birth in a hospital setting is the safest option, (i.e. compared to an out-of-hospital birth); a perception reinforced by the media, most prenatal educational material, and conversations with loved ones (Klein et al., 2006; Miller and Shriver, 2012). In addition, anxiety about pain during delivery can contribute to an increased preference for cesarean birth, epidural anesthesia, and other types of pharmaceutical pain management not available outside of a hospital (Klein et al., 2006; Cheyney, 2008; Caughey and Cheyney, 2019). Unsurprisingly then, the vast majority of births in the United States (98.4%) occur in hospital settings (MacDorman and Declercq, 2019).

Yet growing evidence demonstrates the safety and benefits of “community” care and birth—defined as maternity care and birth experiences physically centered within a community and outside of a hospital (Davis-Floyd and Cheyney, 2019)—for low-risk pregnancies when attended by skilled midwives or (a very few) holistic obstetricians (Cheyney et al., 2014; Hutton et al., 2016; Rossi and Prefumo, 2018). Women seeking community care generally elect to give birth at home or in a freestanding birth center (as opposed to a hospital-based birth center, which is directly affiliated with, and usually inside, a hospital). The freestanding birth center offers a space designed to support women during labor and delivery, while keeping low-risk women out of the hospital (Caughey and Cheyney, 2019). Whether at home or in a birth center, women who choose community birth are attended by a midwife, barring serious complications that require hospital transfer, which occurs in around 11% of planned community births (Cheyney et al., 2014). The majority of midwives attending community deliveries in the United States are Certified Professional Midwives (CPMs), who are trained either by apprenticeship or in vocational midwifery schools, and are required to pass a national exam by demonstrating that they have the knowledge and skills required to attend out-of-hospital/community births (Davis-Floyd, 2018). There are approximately 3,000 practicing CPMs in the United States and its territories (Ida Darragh, NARM Board Chair, personal communication, September. 2020), and 13,024 CNMs—certified nurse-midwives—of whom only around 200 presently attend community births (American College of Nurse-Midwives(ACNM), 2020).

In the past few decades, recognition of the services offered by community midwives has slowly grown, resulting in a small but significant increase in women seeking community births across the United States (MacDorman et al., 2014; Caughey and Cheyney, 2019). Many women report choosing a community delivery because they wish to have more autonomy and control over their birth experience, avoid unnecessary medical interventions, experience provider care continuity, and give birth in what they perceive as a safe, familiar environment (Boucher et al., 2009; Zielinski et al., 2015). The CPMs who primarily attend such births have an approach to maternity care that is explicitly person-centered. “Person-centered care” has been defined in this context as services that account for the values, experiences, and circumstances unique to each individual, while also encouraging the participation of women (and their families) in care decisions (Kozhimannil et al., 2015). Midwifery care aligns well with these goals, usually leading to positive perinatal experiences, including increased comfort discussing care and greater client satisfaction with providers (Kozhimannil et al., 2015; Mattison et al., 2018). In addition, women of color are increasingly seeking community care in order to avoid the structural racism experienced in many hospital settings (Thompson, 2016; Davis, 2019).

Despite the benefits of community care for low-risk women, structural factors limit access to it. Specifically, local availability of care options and socioeconomic position have been shown to influence ability to use preferred providers and care facilities (Miller and Shriver, 2012). Individuals living in areas with few local care options may have to travel farther to access desired care, a pattern also linked with reduced prenatal care (Kitsantas et al., 2012; Meyer et al., 2016). Certain provider and facility types may also be completely inaccessible in some locations. As of 2017, 12 states had fewer than 20 freestanding birthing centers across the entire state, with some states having none (MacDorman and Declercq, 2019). Laws regulating midwifery practice within the United States also vary by state. For example, CPMs can practice legally in only 36 states, though they do practice outside the law in many other states, where they are striving for legalization (Davis-Floyd, 2018). Their questionable legal status in 14 states and low numbers in general potentially limit access to CPMs as primary care providers and curtail some women’s ability to plan a community birth (Suarez and Bolton, 2018).

Geographic barriers to maternity care access are also compounded by socioeconomic factors. For instance, community care and delivery options are often not fully covered by insurance plans, further limiting women’s choices (MacDorman and Declercq, 2019). Home births and birth center deliveries are typically much less expensive than hospital deliveries. The average cost for the full course of perinatal care and an uncomplicated home birth with a midwife is $2,870; for birth centers, the average cost is $7,240; for hospitals, the average cost for an uncomplicated vaginal birth is $12,156 (Anderson, Daviss, and Johnson, 2021). Many insurance plans will cover hospital-based care but not all community care options (MacDorman and Declercq, 2019). One recent study using United States national birth certificate data found that in the year 2017, approximately two-thirds of planned home births and one-third of birth center deliveries were self-paid by the mother, compared to only 3% of hospital deliveries (MacDorman and Declercq, 2019). Thus, a community birth is likely to be cost-prohibitive for many because it would have to be covered out-of-pocket, as is evident among individuals on Medicaid. In 2017, Medicaid covered just 8.6% of planned home births and 17.9% of birth center births, compared with 43.4% of hospital births (MacDorman and Declercq, 2019). Coverage also varies by state, with some states, (i.e. Alaska, Rhode Island, Vermont, and Washington) exhibiting much lower rates, (i.e. under 20%) of home births paid for out-of-pocket (MacDorman and Declercq, 2019). Both geographic and socioeconomic factors, along with provider accessibility, therefore appear to influence individual ability to access preferred care.

Women’s ability to access preferred maternity care providers and facilities is important for improving maternal agency and satisfaction (Peters et al., 2019; Vedam et al., 2019). In addition, provider type can heavily influence birth experience. For instance, midwife use has been linked with reduced fear surrounding birth, increased information sharing by providers, and more individual autonomy (Hildingsson et al., 2019). Care satisfaction has also been shown to significantly impact birth outcomes, such that mothers who report being satisfied with their prenatal care are less likely to use pain relief in labor, deliver healthier babies, and are at lower risk of postpartum depression (Nicoloro-SantaBarbara et al., 2017). Conversely, women reporting dissatisfaction with their maternity care have reported increased pain during delivery. This is a concerning pattern, as poor birth experiences have been linked with elevated risk of postpartum depression (Bell and Andersson, 2016). Since prenatal care satisfaction is essential for optimizing labor and delivery outcomes and maternal mental health, it is important to understand and facilitate maternity care preferences, as well as factors that shape and constrain access to favored providers and facilities.

The complex factors influencing maternity care preferences and care access are currently in flux due to the ongoing COVID-19 pandemic, which has dramatically impacted all aspects of United States maternity care. Partners and support persons, including doulas, have not been allowed to attend prenatal appointments in hospitals and have even been barred from attending deliveries (Davis-Floyd, Gutschow, and Schwartz, 2020; Diamond et al., 2020; Gildner and Thayer, 2020). In many cases, newborns have been separated from mothers with confirmed COVID-19 as part of hospital policy (Davis-Floyd, Gutschow, and Schwartz, 2020; Diamond et al., 2020). Women also report increased fear of viral exposure when attending appointments or laboring in a hospital setting, and may alter their pain management strategy, (e.g. going without an epidural or nitrous oxide) to reduce perceived risk of exposure (Gildner and Thayer, 2020). Cumulatively, these factors have led many women in the United States to consider community care during the pandemic (Davis-Floyd, Gutschow, and Schwartz, 2020; Gildner and Thayer, 2020; Rocca-Ihenacho and Alonso, 2020).

Although preliminary, current evidence suggests that the increase in women preferring community care during the COVID-19 pandemic may be substantial. For example, our previous work using a sample of 1,400 pregnant women living in the United States found that participants displayed a substantially higher preference for community births than the national average before the COVID-19 pandemic (5.4% vs. 1.6%, respectively) (Gildner and Thayer, 2020). Interestingly, this percentage is also relatively high among those 667 participants who had previously given birth. Of those, 3.1% reported a community delivery for at least one of their previous births, but 5.1% of these same women now reported planning for a community delivery (Gildner and Thayer, 2020). These findings suggest that the documented increased preference for community birth is likely at least partly due to the COVID-19 pandemic, although it is unclear whether these altered maternity care preferences will persist beyond this lengthy period of public health crisis.

Additionally, altered care preferences may not be met due to the aforementioned geographic and socioeconomic barriers to preferred community care. Pre-existing care barriers may also be exacerbated during the pandemic. For instance, the current high rates of pandemic-related unemployment are linked with lost health insurance (Gangopadhyaya and Garrett, 2020), potentially inhibiting ability to afford needed maternity care. Reduced provider hours and clinic closures during the pandemic may also prevent women from accessing their preferred care services (Gildner and Thayer, 2020). Given this background, we consider how the COVID-19 pandemic may alter future maternity care preferences, and whether women anticipate barriers to accessing preferred care. Assessing how the pandemic has influenced women’s care preferences will provide insight into the future needs of the United States healthcare system. Our study speaks to this nexus of scientific research and public health policy. Our driving questions are: Has the COVID-19 pandemic impacted future preferences for prenatal care? Specifically, do women express an increased interest in seeking community care during future pregnancies, after the pandemic subsides? Additionally, do women who used community care for the first time during their most recent pregnancy because of the pandemic indicate a preference for community care in future pregnancies, even once the pandemic is under control? What factors or experiences have contributed to altered preferences? And finally, what are the most commonly reported anticipated barriers to accessing preferred care in future pregnancies?

## Materials and Methods

To explore factors influencing altered care preferences and potential barriers to care access, we designed the “COVID-19 And Reproductive Effects” (CARE) study. We posted the CARE study on social media platforms (Facebook, Twitter) and distributed it via email to contacts working in maternity care and public health. Pregnant women over the age of 18 and living in the United States were eligible to participate. In order to increase sample diversity, we shared the study information with Indigenous, Black, and Latinx pregnancy groups, and reached out to contacts working in different geographic regions of the United States Participants who completed the prenatal survey and agreed to be re-contacted received a postnatal survey four weeks after their due date. The postnatal data presented here were collected between June 5–December 15, 2020. This study received ethical approval from Dartmouth College (STUDY00032045). We obtained informed consent from all participants. The survey was administered in REDCap, which automatically captures survey responses. The survey completion rate was 92.9% (1,092/1,175 participants). Survey questions regarding future care preferences were added following the start of data collection, after approximately 100 participants had already filled out the postnatal questionnaire, leading to the completion of 980 questionnaires with these responses.

### Key Variables

*Novel preference for community birth*: A novel preference for community care was defined as women who used community care for the first time during the pandemic and indicated a continued preference for this care model during future pregnancies, as well as women who did not use community care in their most recent pregnancy but intended to during future pregnancies. Participants were asked whether they had changed to a community birth during their most recent pregnancy due to the COVID-19 pandemic. Participants were also asked to select which facility option they would prefer in a future pregnancy from the following list:

- I would want to give birth in the same type of facility I used for this pregnancy;
- I would now want to give birth in a hospital;
- I would now want to give birth in a hospital-based birth center;
- I would now want to give birth in a freestanding birth center;
- I would now want to give birth at home.

For our analysis, individuals were coded as exhibiting a novel preference for community birth (in their most recent or a future pregnancy): 1) if they indicated that they had changed to a community birth during their most recent pregnancy due to the pandemic and also specified that they wanted a community birth in the future, (i.e. they do not plan to switch back to a hospital birth once the pandemic subsides), or 2) if they stated that in the future they wanted to give birth in a freestanding birth center or at home (but had not delivered in these facility types previously, including their most recent pregnancy).

*Reasons for preferring this care option*: Respondents were asked why they would select their listed preferred facility type for future pregnancies. For our qualitative content analysis, we used a conventional approach of open followed by focused coding, (e.g. Hsieh and Shannon. 2005; Saldaña, 2009). Specifically, first author Theresa Gildner read through all participant responses and took notes on keywords or phrases that were repeatedly used. She then generated preliminary codes she shared with co-author Zaneta Thayer, who then reviewed a subset of responses using these codes, adding new codes when no existing code matched the data (Table 1). Two Dartmouth undergraduate research assistants, Amanda Lu and Cecily Craighead, then independently coded the responses. Disagreements were discussed and reconciled between the two coders and Gildner to ensure consistency.

**TABLE 1:** Codes generated based on common themes identified across participant qualitative descriptions of reasons for care preferences during future pregnancies.

Code name	Code description
Trust	Trust this care type based on previous experience, reputation, and/or level of provider training and expertize
Person-centered	Desire for more person-centered care (greater autonomy, provider communication, and more personalized care)
Holistic/natural	More holistic/natural care model used (fewer interventions and less medicalized)
Safety	Desire to feel safe, comfortable, taken care of, and/or less stressed
Pain	Want access to the pain management option(s) available with this type of care
Risk	High-risk pregnancy or previous cesarean section so feel must deliver using this option and/or want access to emergency care in case of complications
Disease exposure	Feel there is a low risk of disease exposure with this care type

*Expected barriers to preferred care during future pregnancies*: Participants were asked if they anticipated any factors limiting access to their preferred facility type in a future pregnancy (Yes/No). If participants selected “Yes,” they also indicated which of the following factors were expected to limit their access (selecting all that applied):

- My preferred care type is not available in my area;
- My preferred care type is not covered by my insurance;
- My preferred care type is too expensive;
- Other, describe.

### Sample Characteristics

As described elsewhere (Thayer and Gildner, 2020), demographic data were collected on participant age, race/ethnicity, household income, education, zip code, and prior birth(s). Rural-Urban Continuum Codes (RUCC) were generated from zip code data to assess local population size (Gildner et al., 2020). Briefly, the RUCC is based on the county located at the zip code center point, with each participant receiving a code reflecting local population size and whether the area is classified as metropolitan or non-metropolitan (USDA, 2013). To assess insurance coverage, respondents were asked if they currently had any form of medical insurance (Yes/No), and whether their current insurance plan covered their preferred maternity care (Yes/No). Additionally, participants reported whether they were on Medicaid (Yes/No). For those participants who had given birth previously, prior birth location was also reported and coded as community (home or freestanding birth clinic) or in-hospital (hospital or hospital-based birth center). Finally, respondents reported where they had given birth during their most recent pregnancy (during the pandemic).

### Analytic Approach

We conducted data analyses using Stata 14, generating sample descriptive statistics and assessing participant responses to address the primary study questions listed above.

*Question 1:* We calculated the frequency of women in the sample reporting a preference for community care during future pregnancies. Additionally, as described above, we coded the reasons given by women for these preferences to identify common themes and assessed the frequency of these coded responses within the sample (Table 1).

*Question 2*: We measured the percentage of participants who reported anticipated barriers to preferred future care, as well as the frequency with which each barrier type was selected from the provided list.

## Results

### Sample Characteristics

Sample descriptive statistics are presented in Table 2. Participants lived in all 50 United States states and the District of Columbia.

**TABLE 2:** Descriptive statistics of study sample. Sample means (with standard deviation and range) or frequency (percent) of model variables, for 980 participants.

Variable	Mean (SD; range)
Age (years)	31.9 (3.99; 18–47)
	Frequency (%)
Race/ethnicity	
White	868 (88.6%)
Hispanic, Latinx, or Spanish origin	48 (4.90%)
Black or African American	10 (1.02%)
Asian	28 (2.86%)
American Indian or Alaskan native	6 (0.61%)
Native Hawaiian or other Pacific Islander	3 (0.31%)
Other	17 (1.73%)
Location	
Metropolitan area, >1,000,000	583 (61.8%)
Metropolitan area, 250,000–1,000,000	209 (22.2%)
Metropolitan area, <250,000	70 (7.42%)
Non-metropolitan area	81 (8.59%)
Household income	
< $49,999	86 (8.86%)
$50,000—$99,999	298 (30.7%)
$100,000+	587 (60.5%)
Education level	
Less than a bachelor’s degree	146 (14.9%)
Bachelor’s degree	356 (36.4%)
Degree beyond a bachelor’s degree	477 (48.7%)
Insurance coverage	
No	6 (0.61%)
Yes	974 (99.4%)
Insurance cover preferred maternity care	
No	48 (4.94%)
Yes	924 (95.1%)
Medicaid coverage	
No	908 (93.3%)
Yes	65 (6.68%)
Previous birth (before pandemic)	
No	496 (50.7%)
Yes	482 (49.3%)
Ever given birth at this location (before pandemic)	
Hospital or hospital-based birth center	464 (96.3%)
Home or freestanding birth clinic	18 (3.73%)
Birth location during the COVID-19 pandemic	
Hospital or hospital-based birth center	939 (95.8%)
Home or freestanding birth clinic	39 (3.98%)
In a car	2 (0.20%)
Novel future preference for community birth	
No	922 (94.1%)
Yes	58 (5.92%)
Anticipate barriers to future care	
No	930 (94.9%)
Yes	50 (5.10%)

### Altered Care Preferences During Future Pregnancies

A total of 58 participants (5.92%) in the sample reported a novel preference for community birth following the onset of the COVID-19 pandemic, compared to 22 participants who had already preferred community care (even prior to the onset of the pandemic)–an over 200% increase in preference for community care. There was a clear overlap between preference for community delivery and midwifery care (51 participants preferred midwifery care, 87.9% of participants had a new preference for community care). Out of the participants exhibiting a novel preference for community care, 18 of the 20 women who reported changing from a hospital to a community birth during the pandemic also indicated they would prefer a community delivery during future pregnancies as well.

Of these 18 participants, 14 indicated they switched because they were concerned about hospital limits on support persons being able to attend delivery, and therefore having to labor alone. Thirteen of these women also reported being worried about being separated from their baby in a hospital setting, while 13 also indicated they were afraid of contracting COVID-19 at the hospital. Another 13 of these women indicated that they were concerned about restrictive hospital policies, (e.g. being forced to wear a mask during active labor). In addition to the 18 participants who chose a community birth for their most recent pregnancy and indicated they would also seek community care during future pregnancies, 40 women reported that they planned to opt for a community birth during future pregnancies (after the pandemic), resulting in a total of 58 participants exhibiting a novel preference for community care. When asked why they would select a community location for future deliveries, some common themes emerged. Of the subset of 53 participants with a novel preference for community care who described the reasons behind this partiality, 34.0% (18/53) stated that they perceived these care options to adhere to a more “natural” birth model, with less reliance on medical interventions or medications to speed up delivery, and more holistic and continuous care throughout pregnancy and the postpartum period. Similarly, 30.2% (16/53) of these participants reported that they felt these options would be more person-centered, (e.g. more effective provider communication, greater respect for the autonomy of the women and her birth plan, and more personalized care). In addition, 11.3% (6/53) of these respondents preferred a community delivery due to a lower perceived risk of pathogen exposure, while 37.7% (20/53) of participants described preferring community care because they felt safe and well cared for in these settings.

Participants who expressed a preference for in-hospital deliveries during future pregnancies were also asked to describe why they favored this option. A total of 900 participants indicated they preferred an in-hospital delivery, (i.e. hospital or hospital-based birth center). Of the 620 participants who described why they preferred an in-hospital birth, 46.5% (288/620) reported a desire to deliver in a hospital to ensure easy access to medical interventions, either due to personal risk factors, (e.g. previous cesarean birth) or in case of complications during delivery. Likewise, 32.6% (202/620) of these respondents stated that they felt most safe and well cared for in a hospital. Medicalized pain management also appeared to be a consideration for some women; 4.5% (28/620) of participants indicated that this was a primary motivation for seeking an in-hospital delivery in the future. Finally, 44.0% (273/620) of these respondents reported preferring an in-hospital delivery because they trusted the experience and training of medical staff, (e.g. due to personal experience in past deliveries or facility reputation).

### Anticipated Barriers to Accessing Preferred Care During Future Pregnancies

Fifty participants (5.1% of the sample) reported that they expected barriers in accessing their preferred future care provider or facility type. Of those reporting a novel preference for community delivery, 34.5% (20/58) stated that they anticipated barriers that could prevent them from accessing their preferred care. We asked these respondents to indicate which factors may inhibit their care access. Seven participants reported that their preferred community care type was not available locally (was located too far away), eight stated that their preferred care type was not covered by their insurance, and eight said that it was too expensive. All other participants reported barriers that could be classified as reflecting high-risk pregnancies; specifically, six women reported they would likely be unable to access preferred care due to underlying medical conditions, age restrictions, or because they had previously delivered by cesarean.

## Discussion: Novel Preferences for Community Care and Barriers to Access

While the overwhelming majority of births in the United States occur within hospital settings, there has been an increase in community births in recent years. Our findings support the idea that the COVID-19 pandemic may have further accelerated this shift in maternity care preferences. These novel preferences were evident both among women who changed their birth plans during the pandemic and among those who were unable to alter their birth plans during this most recent pregnancy, but who stated that they intend to seek community care in future pregnancies. Notably, while the pandemic was the impetus for many women to explore out-of-hospital birthing options, the reasons why women stated that they preferred community births were largely consistent with reasons found in studies prior to the pandemic, including patient-centered care and preference for less medical intervention. Although the majority of these participants did not report any anticipated barriers to accessing their community care preferences, a relatively high proportion (over one-third) did indicate that they expected such limitations. This finding shows that there are perceived and real barriers to community care access in the United States Since our sample was whiter, wealthier, and more educated than the general population, it is likely that the prevalence of barriers would be even higher among a nationally representative sample of birthing mothers, particularly one that included more women of color.

### Pandemic-Related Changes in Care Preferences

Of the 20 women who switched to community care during the pandemic, 18 reported that they would prefer a community birth during future pregnancies (while the other two indicated they would prefer an in-hospital birth in the future), even once the pandemic subsides. This suggests that their community-based perinatal experiences during this most recent pregnancy were positive, reinforcing their desire to use community care going forward (all respondents quoted below are white, reflecting the great majority of our sample). For example, one woman who switched to a community delivery during the pandemic stated:

*Switching to the birthing center and midwife care was a blessing in disguise. They were totally aligned with our birthing goals and helped to facilitate the experience we wanted far better than a hospital and/or our previous provider could have* (33-year-old, primigravid participant with a Bachelor’s degree, living in a metro area of over one million people).

Likewise, a second participant described the benefits resulting from this unexpected birth plan change, affirming her desire to use community care during any future pregnancies:

*Home birth was a wonderful and less stressful experience than my previous two hospital births—simply because I was at home which was a significantly less stimulating environment and I was surrounded exclusively by known people who I have established trusting relationships with* (36-year-old, multigravid participant with a Master’s degree, living in a metro area of over one million people).

Such participant experiences, which are representative of others, highlight how altered birth plans in response to the pandemic may lead to a continued preference for community deliveries in the future. Pandemic-related fears, (i.e. restrictive hospital policies, limited support during labor, separation from their infant, and disease exposure in a hospital setting) appear to have led women to assess the merits of community care options, altering their birth plans in some cases as they learned more about these previously unconsidered options and leading to lasting changes in care preference. While shifts towards community birth existed prior to the pandemic (MacDorman et al., 2014; Caughey and Cheyney, 2019), these results suggest that the pandemic may have served to further accelerate this shift. Several participants described how the pandemic had made them more aware of community care options:

*Home birth was a great experience that I may not have tried without the pandemic* (28-year-old, multigravid participant with a PhD, living in a metro area with a population of 250,000–—one million people).

*Because of Covid and limitations in place, I have educated myself on other options and feel they meet my needs and the type of birth experience I want* (32-year-old, multigravid participant with a Master’s degree, living in a metro area of over one million people).

Some participants went further, describing how the pandemic led them to consider alternative birth models. As one respondent said:

*While I was born at home myself, had not really considered home birth until the COVID-19 pandemic hit and it became possible that hospital facilities could be overwhelmed and my partner would not be permitted to attend me. In considering home birth for those reasons, I also became more concerned by the medicalization of the birth experience that can be associated with a hospital experience* (36-year-old, primigravid participant with a Bachelor’s degree, living in a non-metro area).

Concern about the medicalization of hospital birth was common throughout the subset of women reporting a new preference for community deliveries, as was a preference for a more holistic, natural birth model (34.0% of the subset). Women also asserted that they believed community births to be safer, both because community care prevented pathogen exposure (relative to a hospital setting) and/or because they felt most comfortable delivering in a less-medicalized environment (11.3% and 37.7% of the subset, respectively). Participants also reported a preference for community care due the belief that it is more person-centered (30.2% of the subset), thereby alleviating stress and enhancing their autonomy. As respondents noted:

*I also love the idea of staying in the comfort of home and having a skilled birth [attendant] come to me instead of having to worry about when to leave for the hospital. And I find driving home with a newborn stressful, but birthing at home removes that factor* (37-year-old, multigravid participant with a professional degree, living in a metro area of under 250,000 people).

*Homebirths are AMAZING!!! I got to co-sleep and do what I wanted to do …* (32-year-old, multigravid participant with a Bachelor’s degree, living in a metro area of over one million people).

Nevertheless, as noted above, of the 900 participants who described why they would prefer an in-hospital birth during a future pregnancy, nearly half (46.5%) stated that this was because it afforded ready access to medical interventions in case of complications. Furthermore, in contrast to the women who reported feeling safest outside of a hospital, nearly one-third (32.6%) of the participants preferring in-hospital care described feeling more secure and cared for in a medical environment. These findings cumulatively suggest that most U. S. women still perceive community deliveries as inherently more dangerous than giving birth in a hospital—a concern that could outweigh competing desires to avoid hospital delivery during the pandemic. Yet it is interesting to note that although participants used similar terms (“safe,” “secure,” “less dangerous,” etc.) to describe their preferences, individuals appeared to have very dissimilar perceptions of what these terms could mean in different circumstances and how these concepts applied to maternity care. A subset of women clearly defined “safe maternity care” as more medicalized, with easily available interventions and OB/GYNs. In contrast, other participants considered less-medicalized community care with fewer interventions to be the “safer” option.

These opposing views are likely due to a range of factors, including personal experience, stories from friends and family, exposure to various maternity care models in the media, and knowledge about the benefits and risks associated with each option (Klein et al., 2006; Sunil et al., 2010; Miller and Shriver, 2012; Smith et al., 2018). Many of these proximal factors may reflect the normative acceptance of the biomedicalization of childbirth in United States contexts (Jordan, 1993; Davis-Floyd, 2003, 2005; Wendland, 2007). However, as with so much else during the COVID-19 pandemic, this moment may represent an inflection point for some women, causing them to reassess their previous perceptions regarding the safety of hospital vs. community care. Indeed, the fact that 5.92% of the sample exhibited a novel preference for community deliveries is meaningful. Although birth center births more than doubled and home births increased by 77% between 2004 and 2017 in the United States, only one of every 62 births (1.61%) was classified as a community delivery in 2017 (MacDorman and Declercq, 2019). Thus, the 5.92% novel preference for community births during the pandemic in our sample could represent a substantial increase within the United States birthing population as a whole. These altered preferences may subsequently foster a greater demand for midwifery-led person-centered care models in community settings in the coming years, even after the pandemic ends, leading to better birth outcomes and large cost-savings (Anderson, Daviss, and Johnson 2021).

### Barriers to Accessing Preferred Future Care

Only 50 respondents (5.1% of the sample) indicated that they anticipated barriers in accessing preferred future care types. Yet a relatively high percentage (34.5%) of participants expressing a novel preference for community deliveries reported that they expected barriers in accessing these new care preferences. Reported barriers, (e.g. lack of insurance coverage) may become more common in coming years, as evidenced by recent changes in maternity care costs. Out-of-pocket maternity care costs for all services have risen in the last decade, including for women with employer-based insurance and those in higher-income brackets (Moniz et al., 2020). Specifically, the Affordable Care Act requires employer-based insurance plans to cover maternity care, but plans are allowed to impose high deductibles and copayments for these services.

As a result, out-of-pocket service costs rose between 2008–2015, despite the cost of care remaining the same; a pattern largely attributed to rising deductibles (Moniz et al., 2020). These higher costs may lead women to delay or avoid needed care, which could subsequently lead to poor maternal and infant health outcomes. Thus, while the relatively affluent women in this sample may have been able to afford these higher out-of-pocket costs, evidence suggests a current trend of rising service costs for all women living in the United States–-a pattern that is especially concerning given the growing unemployment rates, lost insurance coverage, and financial stress associated with the COVID-19 pandemic. If out-of-pocket maternity care costs continue to increase as the economic consequences of the pandemic persist, it seems likely that a larger portion of the pregnant women living in the United States will be unable to access preferred and needed services. This will be particularly evident for lower income women, who are more likely to be women of color.

### Medical Care and Policy Implications

Even prior to the pandemic, United States maternity care outcomes were troubling. Recent estimates indicate that the United States spends roughly 17.8% of national gross domestic product (GDP) on health care, significantly higher than other high-income nations, which tend to range between 9.6 and 12.4% (Papanicolas et al., 2018; Martin et al., 2019a). Despite this high investment in health care, the United States consistently reports worse health outcomes relative to other high-income countries, with fewer people accessing health insurance, lower life expectancies, and higher maternal and infant mortality rates (especially among Black women) (Gunja et al., 2018; Papanicolas et al., 2018). These poor outcomes have led to active calls for healthcare reform in the United States. (e.g. an increased interest in “Medicare for all”)--demands expected to be bolstered by the economic and medical fallout of the COVID-19 pandemic (King, 2020).

There are already signs of shifts in medical care services and insurance coverage. To take one example, Medicaid and other insurance types have recently expanded coverage to include telehealth appointments–including for maternity care–during the pandemic (Fryer et al., 2020). These changes are likely to also impact access to maternity care options, potentially enhancing the availability of community care services. For instance, in June 2020 the New York State COVID-19 Maternity Task Force announced that New York Governor Andrew Cuomo had directed the State Department of Health to allow freestanding birth centers run by midwives to operate independently for the first time in state history (New York State Government, 2020), providing pregnant women in New York State with more care options to meet their specific needs. Moreover, the task force moved to expedite the licensure process required to certify midwife-led freestanding birth centers (New York State Government, 2020).

Similar efforts to expand access to community births and midwifery care during the pandemic are also evident in other states, (e.g. Maine, New Jersey, Pennsylvania, Tennessee, and Texas) (Platt, 2020). These changes may have the added benefit of addressing the anticipated shortage of OB/GYN providers expected in coming years as a high proportion of doctors retire amidst a national shortage of younger doctors. This expected shortage has been attributed to a range of factors, including fewer ON/GYNs providing around-the-clock care and more maternity care doctors practicing subspecialties that do not involve routine deliveries (Ollove, 2016). More readily available midwifery community-based care may consequently address expected rising national demands for maternity care providers.

### Study Limitations

Important study limitations exist. First, as previously mentioned, these data are not representative of the United States population as a whole. Women in our sample were older (31.9 years vs a national average of 29.0 in 2018), more likely to be white (88.6% in this sample vs 51.6% nationally), highly educated with at least Bachelor’s degree (85.1% vs 33.0%), and were slightly more likely to live in a metropolitan area (91.4% vs 86.5%) (Centers for Disease Control and Prevention (CDC), 2020; Martin et al., 2019b). These demographic differences may contribute to variation in community care preferences and access. Evidence indicates that lower education levels are associated with limited knowledge of all care options (Sunil et al., 2010; Smith et al., 2018), resulting in higher rates of community births among well-educated women (Boucher et al., 2009), who are also more likely to be able to afford them. The high education levels evident in this sample may predispose participants to consider and seek out community care options.

In addition, rates of community birth are higher among white women than women of color (MacDorman and Declercq, 2019). This pattern has been attributed to a range of reasons, including financial barriers to preferred care and racial and ethnic disparities in high-risk pregnancy diagnoses, (e.g. preeclampsia and diabetes) that may increase the likelihood of delivery in a hospital setting for women of color (Howell, 2018; Onwuzurike et al., 2020). Still, the potential value of community births for women of color in particular is being increasingly recognized, as culturally centered community care can reduce exposure to structural racism and customize care to the needs of the individual (Hardeman et al., 2020; Tilden et al., 2020). Future care preference alterations should consequently be explored in more diverse study populations, which may experience greater barriers to learning about and accessing community care, especially during the pandemic.

For example, women of color appear more likely to experience pandemic-related maternity care disruptions for a variety of reasons linked with underlying structural inequities. These factors include less reliable access to phone and internet services needed for telehealth appointments, a greater reliance on public transportation to access care (which may be cut back due to the pandemic), and an increased likelihood of working in essential services (which may curtail their flexibility in scheduling healthcare appointments) (Onwuzurike et al., 2020). It is therefore necessary to consider how pandemic-related care disruptions and healthcare policy changes may exacerbate existing inequities, particularly among minority communities that have historically experienced inferior maternity care, less provider information sharing, and poorer birth outcomes (Niles et al., 2020; Onwuzurike et al., 2020), ultimately diminishing women’s autonomy and their direct involvement in healthcare decisions (Altman et al., 2019). These disparities may consequently influence women’s care preferences and the barriers they may face to accessing their preferred provider or facility.

Another study limitation is that we did not explicitly ask about home-to-hospital transfers; therefore, we do not have data on how many of our participants who planned community births ended up transferring to the hospital. This is an important issue, as other data show that while unforeseen complications are a primary driver of hospital transfers (Caughey and Cheyney. 2019), childbearers who are not fully ideologically committed to home birth do sometimes transfer to hospitals during labor, primarily for labor dystocia/failure to progress, because they feel safest in hospitals (Davis-Floyd, Gutschow, and Schwartz 2020).

## Conclusion: Anticipating Future Needs

As women reassess their birth plans in response to pandemic-related concerns and limitations, it appears that some are learning more about community care options, with implications for current and future decisions. Our findings suggest that positive community delivery experiences during the pandemic, negative perceptions of in-hospital services, and a greater appreciation for the benefits of person-centered care may all contribute to shifting preferences among at least a subset of women living in the United States, and that these altered care preferences may persist beyond the COVID-19 pandemic. If true, this social shift will likely necessitate greater investment in CPM training, legalization, and licensing, as well as expanded insurance coverage to include community care services. Greater availability of community, person-centered care models for low-risk pregnancies may also represent a cost-effective strategy for reducing the current relatively high rates of maternal and infant mortality. Specifically, community care is linked with lower rates of poor birth outcomes, (i.e. preterm birth, low birth weight infants, and neonatal death) (Vedam et al., 2018). The maternity care experiences and preferences of women during the COVID-19 pandemic may therefore offer a view into how care decisions are changing in response to novel conditions, with implications for anticipating and responding to future needs.

## Data Availability

The raw data supporting the conclusions of this article will be made available by the authors, without undue reservation.
